# Chang’E-6 reveals solar wind–dependent H^−^ ions on the Moon

**DOI:** 10.1126/sciadv.adw1162

**Published:** 2026-03-13

**Authors:** Tianhua Zhong, Lianghai Xie, Aibing Zhang, Martin Wieser, Wenjing Wang, Mats Holmström, Romain Canu-Blot, Lei Li, Stas Barabash, Yongliao Zou, Yiteng Zhang, Qi Yan, Weibin Wen, Xiangjin Deng, Hongqian Cao, Chi Wang

**Affiliations:** ^1^ National Space Science Center (NSSC), Chinese Academy of Sciences (CAS), Beijing 100190, China.; ^2^ College of Earth and Planetary Sciences, The University of Chinese Academy of Sciences, Beijing 101408, China.; ^3^ The University of Chinese Academy of Sciences, Beijing 101408, China.; ^4^ Swedish Institute of Space Physics (IRF), Kiruna, 98192, Sweden.; ^5^ National Astronomical Observatories, Chinese Academy of Sciences, Beijing 100101, China.; ^6^ Beijing Institute of Spacecraft System Engineering, Beijing 100094, China.

## Abstract

Apart from positive ions and electrons, negative ions are expected in various astrophysical environments. However, they have never been detected on the Moon until the Chang’E-6 mission. The NILS instrument onboard Chang’E-6 lander is the first dedicated instrument for detecting negative ions beyond Earth and has successfully obtained H^−^ spectra on the lunar surface, providing an unprecedented opportunity to investigate their origin and distribution. Here, we present a positive correlation between the H^−^ spectra and solar wind parameters, which provides direct evidence for the generation of negative ions from solar wind–surface interaction. Combined with Monte Carlo simulations, we predict a thin dayside H^−^ layer and a long nightside H^−^ tail, which can contribute to the lunar plasma environment, especially during an extreme solar wind density event. These findings greatly improve our understanding of the generation and distribution of negative ions on the Moon and other airless bodies.

## Teaser

Solar wind–derived negative ions have been confirmed on the Moon, which open a window for space plasma and planetary science.

## INTRODUCTION

More than 99% of the visible matter in the universe exists in the plasma state that is normally thought to be made up of positive ions and electrons. However, negative ions are also expected to be present in space plasma, which can play crucial roles in various astrophysical environments (*1*). In the Sun’s outer layers, H^−^ ions dominate the visible opacity (*2*). In addition, H^−^ ions may be critical to the formation of the early Universe’s structure by enabling the rapid generation of molecular hydrogen through associative detachment reaction. This process acts as an effective coolant, allowing gas masses to collapse and form the first generation of stars (*3*, *4*). Astronomical observations and models have shown that negative ions can accumulate to high densities within interstellar clouds, prestellar cores, and protostellar envelopes, substantially influencing the chemical processes and evolution of these regions (*5*, *6*).

Negative ions can be generated via both gas-phase and surface processes (*1*). Gas-phase processes, like radiative electron attachment and dissociative electron attachment, are important for the generation of negative ions in planetary ionospheres and interstellar clouds. Previously, negative ions in planetary ionospheres were identified by several missions primarily using data from electron spectrometers (ELS). The ELS onboard the Giotto spacecraft detected negative ions of various mass groups in the coma of comet Halley (*7*). Similarly, the Rosetta spacecraft found negative hydrogen ions in the solar wind near the nucleus of comet 67P, which may be produced by the interaction between the solar wind and cometary neutrals (*8*). On Mars, negative hydrogen ions were observed by the ELS of the MAVEN spacecraft, with an H^−^ to H^+^ abundance ratio on the order of 1:10 (*9*). On Jupiter’s moon Europa, negative chlorine ions can be inferred from the wave polarization observed by the Galileo spacecraft (*10*, *11*). In the Saturn system, the Langmuir probe onboard the Cassini spacecraft found heavy negative ions in both Saturn’s and Titan’s ionospheres (*12*–*14*). In addition, the ELS of the Cassini spacecraft detected fluxes of negative ions in the plumes from Enceladus, likely negatively charged water group cluster ions associated with the plume (*15*).

The surface processes dominate the source of negative ions on airless bodies, such as our Moon. In particular, the surface of airless bodies can directly interact with the ambient plasma, like solar wind, which bring negative ions through sputtering and scattering (*16*–*20*). During this process, the neutral particles released from the surface may capture an electron from the ambient atoms with an efficiency that depends on the surface’s work function, as well as the species and ejection velocity of the particles (*18*). In addition, the negative ions may be also generated via electron-stimulated desorption (ESD) or photon-stimulated desorption, as well as micrometeoroid impacts (*1*, *17*, *21*). For instance, photoelectrons emitted from the porous regolith may interact with hydrogen-bearing species adsorbed on the surface—such as molecular hydrogen, hydroxyl groups, and water molecules—to form H^−^ ions. Laboratory experiments have confirmed that ESD can produce H^−^ from a lunar soil simulant (*22*). Therefore, the presence of negative ions is expected on all kinds of airless bodies such as Mercury, moons, and asteroids.

Previously, the Cassini spacecraft’s ELS observed negative oxygen and carbon-based ions near Rhea and Dione, which can be traced back to the vicinity of their surfaces, possibly originating from plasma-surface interactions (*23*–*25*). Our Moon is a typical airless body and can be treated as a natural laboratory to verify the generation of negative ions by the plasma-surface interaction in space. The Moon spends most of its time in the solar wind, and secondary particles with different charge states (neutral, positive, and negative) can be released from the lunar surface due to the scattering and sputtering by the solar wind. Previously, energetic neutral atoms (ENAs) and positive ions emitted from the lunar surface have been observed by Chandrayaan-1 and Kaguya missions in orbit (*26*–*29*) and by Chang’E-4 mission on the lunar surface (*30*, *31*). However, although some orbital missions have an electron detector, the expected negative ions have not been observed by them on the Moon so far. This absence is likely due to the photodetachment effect at 1 astronomical unit (AU) (*32*), resulting in a short lifetime for the negative ions and limiting their average travel distance to much less than the lunar orbit altitude. Moreover, it is not easy to distinguish negative ions from electrons in an electron detector, due to the relatively high concentration of electrons around the Moon.

Compared to the orbital measurements, in situ measurements on the lunar surface can minimize the photodetachment effect and may obtain the maximum negative ion flux. Moreover, the lunar surface is the source region for the generation of negative ions, and hence we can get the original features of the negative ions, such as the energy spectra and the angular distribution, via the surface in situ measurements (*33*). Therefore, surface measurements are particularly important for revealing how negative ions are generated on the Moon as well as other airless bodies. With these in situ measurements, we can further evaluate how negative ions can influence the space environment of the Moon. Furthermore, the energy spectra of these scattered and sputtered particles contain a wealth of information related to plasma-regolith interactions, which play a key role in the formation of solar wind–derived water and in the space weathering processes. Consequently, the measured spectra may also reflect intrinsic surface properties such as composition, porosity, and work function.

The Negative Ions at the Lunar Surface (NILS) instrument onboard the Chang’E-6 lander is the first dedicated instrument for directly detecting negative ions on the lunar surface (*33*), and a distinct H^−^ signal has been successfully detected by NILS, with a differential number flux up to about 4 × 10^3^ cm^−2^ sr^−1^ eV^−1^ s^−1^ (*34*). This work will further check the dependence of the measured H^−^ spectrum on the solar wind parameters and then verify whether the negative ions are from the solar wind–surface interaction. In particular, the dependences of the integrated H^−^ flux and the average H^−^ energy on the solar wind flux and the solar wind energy will be investigated. In addition, the spatial distribution of H^−^ ions and its influence on the lunar plasma environment are quantitatively analyzed via test-particle simulations with the H^−^ yield measured by NILS.

## RESULTS

### H^−^ ions generated from solar wind–lunar surface interaction

**Fig. 1.**
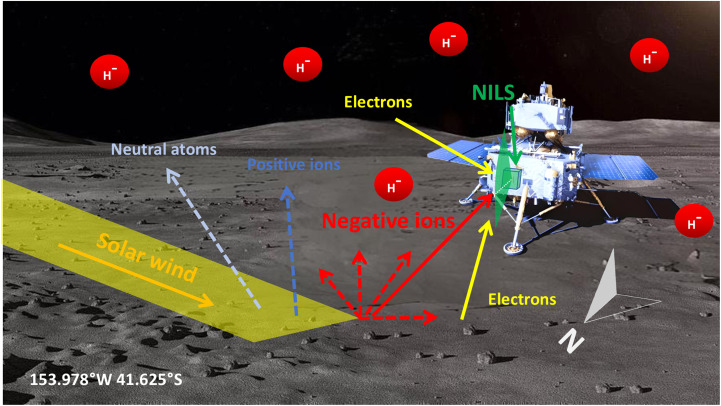
The observation geometry of the NILS instrument onboard Chang’E-6. The green arrow illustrates the NILS instrument, with a field of view directed to the north and an elevation angle range of 120°. The yellow stripe indicates the flow of incident solar wind, while the red arrows represent scattered negative ions. The dark blue arrow represents reflected positive ions, and the gray arrow represents the scattered neutral atoms. The red circles indicate negative ions in the near-Moon space.

**Fig. 2.**
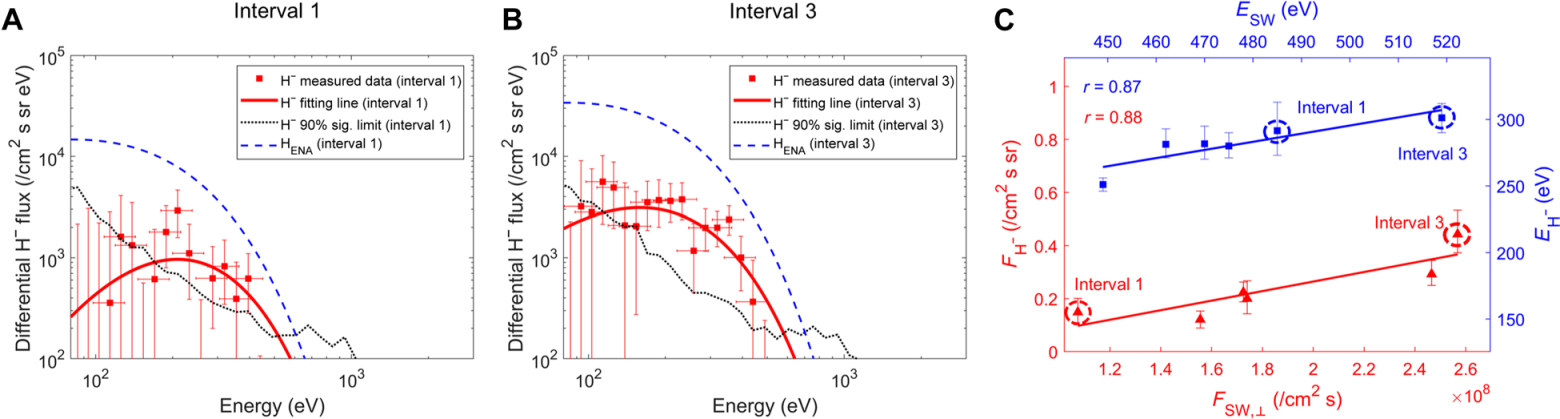
The characteristics of H^−^ spectra observed by NILS. (**A** and **B**) H^−^ spectra and the H ENA spectra under two different solar wind conditions [the observed spectral points were derived using the methods and code provided by Wieser *et al.* (*34*)]. The solid red lines represent the best fits to the H^−^ spectra for intervals 1 and 3, respectively, with the measured data shown as red squares. The vertical error bars represent 68% confidence intervals, and the horizontal error bars represent the energy resolution of the instrument. Confidence intervals without a data point indicate that the data point is below the differential flux range shown. The dashed blue lines indicate the H ENA spectra predicted by the empirical function of Futaana *et al.* (*39*) with the solar wind parameters of intervals 1 and 3, respectively. The dotted line corresponds to a 90% significance limit, below which the differential flux cannot be significantly distinguished from zero. (**C**) Dependences of the integrated H^−^ flux (*F*_H_^−^) and the average H^−^ energy (*E*_H_^−^) on the normal component of solar wind flux (*F*_sw,⊥_) and the solar wind energy (*E*_sw_), respectively. The red triangles represent the *F*_H_^−^, while the blue squares represent the *E*_H_^−^. The vertical error bars represent 68% confidence intervals. The red and blue solid lines show the best linear fits for the flux and energy data, respectively. The observations for intervals 1 and 3 are marked by dotted circles.

At 22:23 UTC on 1 June 2024, the Chang’E-6 successfully landed on the far side of the Moon in the South Pole-Aitken basin (153.978°W, 41.625°S) (*35*). Figure 1 indicates the observation geometry of the NILS instrument, with its field of view oriented in the north direction. NILS began to work shortly after landing and intermittently collected nearly 4 hours of valid data between 03:01 UTC on 2 June 2024 and 03:38 UTC on 3 June 2024. Within this period, significant H^−^ signals that continuously exceed the significance limit curve (which corresponds to a 90% significance limit below which the differential flux cannot be distinguished from zero) were identified in the 80- to 400-eV energy range (*34*). There were six observation intervals longer than 20 min, in which significant counts were recorded by NILS to obtain effective spectra of the H^−^ ions (fig. S1). To verify whether these negative ions are related to the solar wind, we check the dependences of the H^−^ spectra on the solar wind energy and the solar wind flux in the surface’s normal direction. With the measurements from ARTEMIS spacecraft (*36*), we obtain the number density and energy of the solar wind during the same period (table S1), and then we can calculate the normal component of solar wind flux (*F*_sw,⊥_) and the solar wind energy (*E*_sw_) for each NILS interval. It is found that interval 3 has the highest *F*_sw,⊥_ of about ~2.57 × 10^8^ cm^−2^ s^−1^, while interval 1 has the lowest *F*_sw,⊥_ of ~1.07 × 10^8^ cm^−2^ s^−1^. First, we compare the H^−^ spectra between interval 1 and interval 3, with nearly identical observation durations of 23 and 24 min, respectively. As shown in Fig. 2 (A and B), the H^−^ flux of interval 3 is about three times higher than that of interval 1, indicating that there is a positive correlation between the H^−^ flux and the solar wind flux. In addition, both of the H^−^ fluxes are concentrated between 80 and 500 eV, with a peak between 150 and 300 eV. In addition, the cutoff energies (the critical energy where the energy spectrum falls below the significance limit curve) of interval 1 and interval 3 are 395 and 440 eV, respectively. According to the ARTEMIS measurements, the solar wind energies of these two intervals are 485 and 515 eV, respectively. Previously, Chang’E-4 observations found a strong positive correlation between the cutoff energies of the backscattered ENAs and the incident solar wind energies (*37*, *38*). Consequently, our result implies that the H^−^ energy may also be positively dependent on the solar wind energy.

To further check the dependences of observed H^−^ ions on the solar wind parameters, we calculated the integrated flux (*F*_H_^−^) and the average energy of the H^−^ spectra (*E*_H_^−^) above 200 eV for all six intervals and checked their dependences on the solar wind flux and solar wind energy. As shown in Fig. 2C, there is a positive linear correlation with a correlation coefficient of about 0.87 between the *F*_H_^−^ and the *F*_sw,⊥_. Furthermore, although the solar wind energy varies little between intervals, we still find a good linear correlation between the *E*_H_^−^ and the *E*_sw_, with a correlation coefficient of 0.88 (Fig. 2C). The H^−^ ions measured by NILS exhibit strong dependencies on solar wind flux and energy, implying that those H^−^ ions are associated with the solar wind–surface interaction. Furthermore, the average energy of the H^−^ ions is about 250 to 300 eV, suggesting that these H^−^ ions are mainly generated by the solar wind backscattering from the lunar surface, rather than the sputtering. Consequently, our results provide direct evidence for the generation of H^−^ ions from the solar wind–surface interaction.

Previously, similar dependences on the solar wind were also found for the backscattered H ENAs (*39*–*41*). Here, we further examine the difference between backscattered H^−^ ions and H ENAs to better understand the negative ion formation process. Laboratory and theoretical studies have shown that the probability of a particle leaving a surface as a negative ion strongly depends on the perpendicular component of its ejection velocity (*16*, *18*, *42*, *43*). Specifically, a lower perpendicular ejection velocity implies a longer interaction time within the surface, which increases the probability that the loosely bound extra electron will tunnel back to the surface, thereby neutralizing the negative ion. This effect, where the survival probability of negative ions decreases at lower ejection velocities, is referred to as the “ejection-velocity dependence.” In contrast, neutral atoms escaping the surface are not subject to this specific electron-loss mechanism. Therefore, comparing the H^−^ spectra with the H ENA spectra obtained by Chandrayaan-1 provides a way to test whether the observations are consistent with the expected ejection-velocity dependence for negative ions emitted from the lunar surface. Figure 2 (A and B) shows the measured energy spectra for H^−^ ions. When above the peak energy (around 200 to 300 eV), the H^−^ differential flux clearly decreases, similar to what is observed in H ENA spectra. But when below 200 eV, the H^−^ differential flux exhibits a plausible decline toward the lower energy range, suggesting that the H^−^ ions may be more concentrated in the mid-energy range compared with typical H ENA spectra. Such a behavior would be consistent with the “ejection-velocity-dependence” theory. However, the large error bars make the low-energy range statistically uncertain, and hence more precise measurements are needed to confirm this ejection-velocity dependence.

### Distribution of H^−^ ions around the Moon

It is interesting to study the distribution characteristics of H^−^ ions around the Moon and assess their influence on the lunar space environment. Here, we use a test-particle Monte Carlo simulation to investigate the distribution of H^−^ ions around the Moon, based on the NILS measurements. The Selenocentric Solar Ecliptic coordinate system is used, in which the *x* axis points from the Moon’s center to the Sun, the *z* axis is normal to the ecliptic plane, and the *y* axis completes the right-handed set of axes. We simulate five distinct cases to investigate the H^−^ distribution under different solar wind conditions. Two cases are based directly on the observational data from intervals 1 and 3. For interval 1, the solar wind has a density of 6.91 cm^−3^, a velocity vector of (−304.51, 15.75, −8.46) km/s, an interplanetary magnetic field (IMF) of (3.44, −0.69, 1.25) nT, and a temperature of 17.64 eV. For interval 3, these parameters are changed to 15.38 cm^−3^, (−314.80, −1.96, 20.87) km/s, (−4.76, 1.83, −0.63) nT, and 21.90 eV, respectively. The remaining three represent extremely high-density (HD) solar wind cases, which could occur during either Coronal Mass Ejections or Stream Interaction Regions (*44*, *45*). When setting the parameters for these HD cases, the velocity (−315, 0, 0) km/s is specifically chosen to be consistent with the solar wind conditions during interval 3, as solar wind velocity can directly influence the H^−^ energy spectrum. The other parameters—a high solar wind density (60 cm^−3^), a temperature of 10 eV, and an IMF strength of 10 nT—were chosen to fall within the typical range for these extremely HD events (*44*, *46*, *47*). The three HD cases differ only in their IMF orientations, which are set to (10, 0, 0) nT, (0, 10, 0) nT, and (10/$\sqrt{2}$, 10/$\sqrt{2}$, 0) nT. Accordingly, these three cases are hereafter referred to as HD Bx, HD By, and HD Bx&By, respectively.

At the beginning of the simulation, individual H^−^ ions are randomly emitted from the lunar surface, according to the energy distribution and the backscattering yield of H^−^ ions obtained from the NILS measurements. For the H^−^ energy distribution, we propose an empirical function that incorporates dependencies on both the solar wind and the ejection velocity, as described in detail in Materials and Methods. For the backscattering yield, we use a value of 2.5% as reported by Wieser *et al.* (*34*). Previously, observations have shown that the H^+^ ions have a similar angular distribution to that of backscattered H ENAs (*48*), suggesting that the charge state may be not so important for the angular distribution of the backscattered solar wind particles. Because of the limited angular coverage of the NILS observations, the angular distribution of backscattered H^−^ ions is unavailable now. Consequently, here, we assume that the backscattered H^−^ ions have the same angular distribution as the backscattered H ENAs, which can be obtained from Chandrayaan-1 observations (*49*, *50*). Once the H^−^ ions are ejected from the surface, they can be picked up by the solar wind, owing to the Lorentz force associated with the ambient electromagnetic fields. Here, we adopt the electromagnetic fields obtained from a hybrid model (*51*) (see Materials and Methods for details), in which both the magnetic field and the electric field can be enhanced in the lunar wake (*52*). The corresponding results are shown in the Supplementary Materials. For the photodetachment effect on H^−^ ions, we adopted a photodetachment rate of 14.3 s^−1^, based on Desai *et al.* (*32*). Note that solar wind protons not only affect the lunar dayside surface but also reach the lunar nightside surface due to the effects such as the thermal motion and the ambipolar diffusion (*53*, *54*). In this study, we refer to the measurements of the normalized differential ENA flux originating from solar wind plasma interactions with the lunar nightside surface, as reported by Vorburger *et al.* (*55*). We take into account solar wind protons affecting the lunar nightside surface with solar zenith angles (SZA) up to 120°. The photodetachment effect in the shadow of the Moon is not considered, since there is no sunlight in this region.

**Fig. 3.**
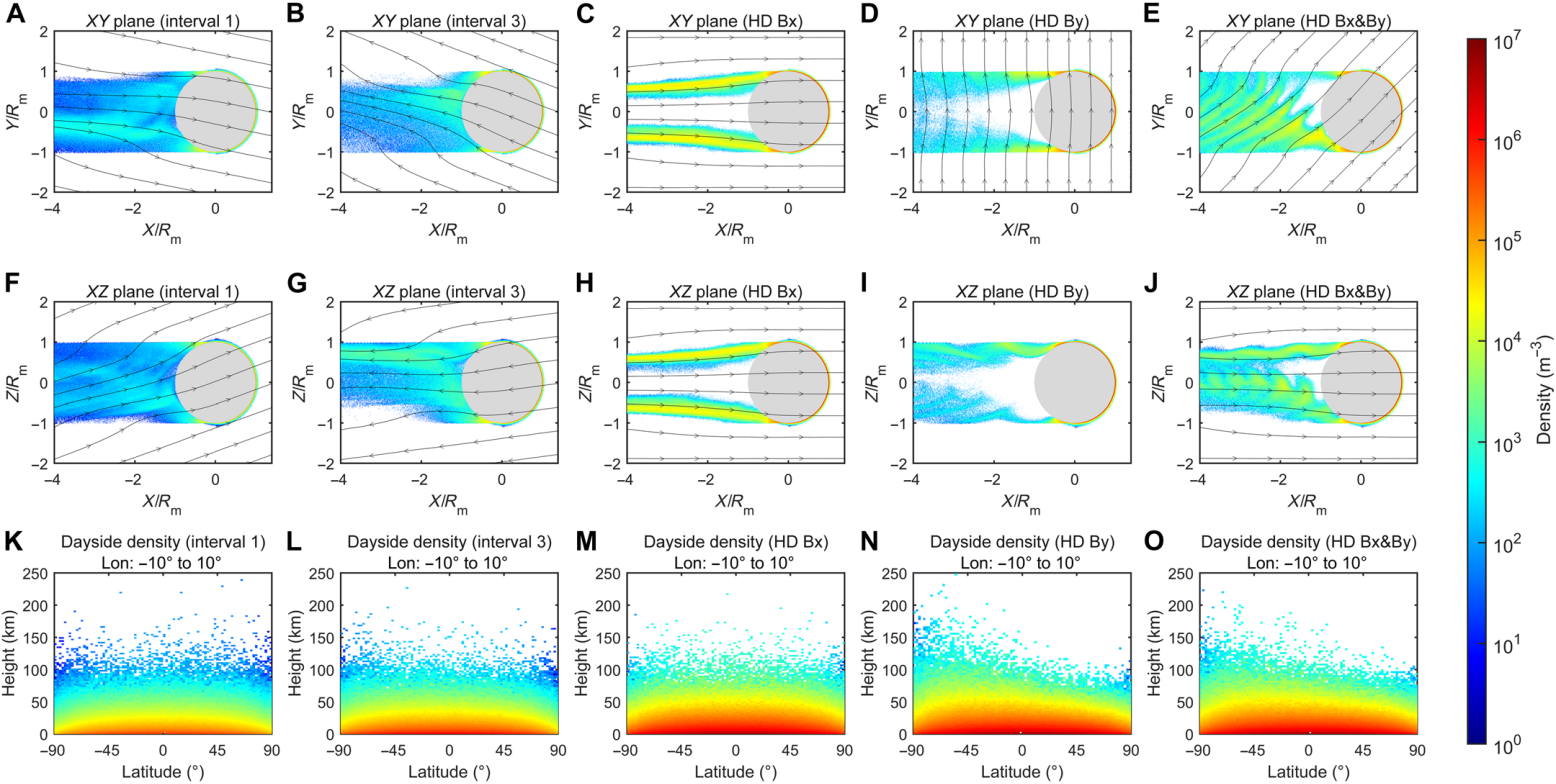
Simulation results of the H^−^ number density in different planes. (**A** to **E**) Density distributions of H^−^ ions in the *XY* planes, for interval 1, interval 3, and the three extremely HD solar wind cases (HD Bx, HD By, and HD Bx&By), respectively. (**F** to **J**) Density distributions of H^−^ ions in the *XZ* planes, for interval 1, interval 3, and the three HD high solar wind density cases (HD Bx, HD By, and HD Bx&By), respectively. The number densities are obtained by counting all simulated particles within ±0.1 lunar radii from the plane. The black lines with arrows indicate the magnetic field lines. (**K** to **O**) Zoomed-in views of the H^−^ density distributions on the dayside, in which the horizontal and vertical coordinates represent latitude and height, respectively. The number densities are obtained by counting all simulated particles within ±10° from the plane.

Figure 3 shows the simulation results of H^−^ number densities in the *XY* and *XZ* planes, within a thickness of ±0.1 lunar radii. First, we can see a distinct negative ion tail on the lunar nightside. For the cases of interval 1 and interval 3, the number density of H^−^ ions in the wake is on the order of 10^3^ m^−3^. Nevertheless, the H^−^ density can approach ~10^4^ m^−3^ near the nightside lunar surface. Previous simulation studies have shown that there is a plasma void in the near-Moon wake region, where the proton density can be smaller than 1% of solar wind density at a downstream distance of about 4 lunar radii and close to zero when near the nightside lunar surface (*56*). Our results suggest that the plasma void may be not so empty due to the presence of H^−^ ions. Furthermore, some H^+^ ions may be attracted into the void region due to the electrostatic attraction of the H^−^ ions. In other words, the H^−^ ions can help to refill the plasma void in the lunar wake. For the HD cases that include a Bx component (i.e., HD Bx and HD Bx&By), the H^−^ number density can reach ~10^4^ to 10^5^ m^−3^ in a large portion of the tail, even at a distance of 4 lunar radii downstream from the Moon. For the HD cases that include a By component (i.e., HD By and HD Bx&By), the H^−^ ion tail exhibits a ribbon-like feature, along with some north-south asymmetries.

The electromagnetic fields play a dominant role in governing the morphology and density distribution of the H^−^ tail, via the Lorentz force. When the magnetic field is primarily aligned with the *x* direction and the corresponding solar wind convection electric field is weak (i.e., interval 1, interval 3, and HD Bx), H^−^ ions predominantly follow the magnetic field lines. But when the magnetic field is perpendicular to the solar wind flow (i.e., HD By case), the H^−^ ions can move in a gyration way, driven by the solar wind convection electric field. Consequently, we can find a ribbon-like tail downstream from the Moon. Moreover, the H^−^ ions can initially move in the −*z* direction, since the solar wind convection electric field is in the +z direction. As a result, it is easier for those H^−^ ions emitted from the Southern Hemisphere on the nightside to get outside of the lunar shadow region, which then disappear due to the photodetachment effect and bring a density loss in the −*z* region. In the HD Bx&By case, H^−^ ions drift not only along the −*x* direction but also in the +*y* direction due to the presence of a Bx component. As a result, the north-south asymmetry observed in the *XZ* plane is different from that in the HD By case (Fig. 3J). For interval 1 and interval 3, as the magnetic field lines are mainly along the *x* direction with a minor perpendicular component, the ion drift speed is relatively low. Moreover, the IMF field strengths of intervals 1 and 3 are relatively weak, which results in a relatively larger ion gyroradius. As a result, both the ribbon-like feature and the near-wake plasma void are less distinct for intervals 1 and 3.

On the dayside, the H^−^ ions are limited in a thin layer near the lunar surface, due to the photodetachment effect, with their density gradually decreasing as the height increases (Fig. 3, K to O). In addition, the negative ion density decreases with increasing latitude because the solar zenith angle is larger at higher latitudes, resulting in a lower solar wind flux in the surface normal direction. A clear north-south asymmetry also exists on the dayside especially for the HD Bx&By and HD By cases. This arises from the gyration of H^−^ ions in the −*z* direction, and the extent of the asymmetry depends on the *z*-component of the convective electric field, which is proportional to the *y*-component of the IMF. For interval 1 and interval 3 cases, the surface density near the subsolar point is approximately 4.5 × 10^5^ m^−3^ and 1.1 × 10^6^ m^−3^, respectively. For the HD case, the surface density can exceed 4.3 × 10^6^ m^−3^, which is approximately 10 times higher than the surface density for the interval 1 case. In addition, the density can fall below 1 × 10^5^ m^−3^ above 50 km under normal solar wind conditions (intervals 1 and 3). This may be the reason why lunar orbiters have not observed any signs of negative ions so far, although the orbiter has an electron detector. However, during an extreme event, the solar wind density will be enhanced, which can bring a larger H^−^ density with a higher height. As shown in Fig. 3O, the H^−^ density can be as large as 1 × 10^5^ m^−3^ at a height of about 40 km, which can provide guidance for the future orbital detection of H^−^ at the Moon.

## DISCUSSION

With the NILS measurements of the Chang’E-6 mission, we investigate the characteristics of H^−^ ions on the Moon. First, it is found that both the flux and the energy of the H^−^ ions show a positive correlation with the flux and the energy of solar wind, respectively, consistent with the generation of negative ions from solar wind–surface interaction. In addition, the H^−^ spectra appear to show lower flux than the H ENA spectra at lower energies, which could be explained by a dependence on the ejection velocity. Combined with Monte-Carlo simulations, we obtain the density distribution of the H^−^ ions around the Moon, consisting of a thin layer (*17*, *34*) on the dayside and a long tail on the nightside. In addition, a north-south asymmetry as well as a ribbon-like tail can be caused by the convection electric field of solar wind. Further studies show that such a H^−^ exo-ionosphere is controlled by solar wind parameters. A higher solar wind density can bring a higher negative ion density, while a higher solar wind speed can increase the height of the negative ion layer. In HD cases, the H^−^ density can be more than 10 times higher than that under typical solar wind conditions. In all cases, the distribution of the H^−^ exo-ionosphere is also controlled by the direction of IMF. In summary, our results reveal the presence of solar wind–dependent H^−^ ions on the Moon.

Negative ions are a newly discovered charged species for the lunar exo-ionosphere (*34*). These H^−^ ions should be nonnegligible for the formation of the near-surface plasma sheath and the lunar wake. In particular, the negative ions can help to refill the plasma void in the lunar wake and to maintain the quasi-neutrality condition in this region. As a result, a full particle-in-cell (PIC) model that can include these H^−^ ions is needed to study the formation of lunar wake. However, such a global full PIC simulation would be computationally infeasible (*51*) and beyond the scope of this work. In addition, these negative ions may bring some special plasma waves or instabilities, due to their different properties (mass, charge, velocity, etc.) from the ambient plasma (protons and electrons) (*57*–*59*). A full PIC simulation is needed to investigate the properties of the waves or instabilities caused by the H^−^ ions, which can then help to search for these waves in the observations. Apart from the direct contribution to the exo-ionosphere, the negative ions may also introduce some new compounds to the neutral exosphere via some special chemical reactions (*1*). For hydrogen negative ions, laboratory results have shown that they can react with neutral hydrogen to produce hydrogen molecules through associative detachment (*60*). Hydrogen molecules have been detected in the lunar exosphere through various methods (*61*, *62*), and this reaction may be one of the sources contributing to the hydrogen molecules of the lunar exosphere. Moreover, they may also react with neutral oxygen through associative detachment to produce hydroxy (*63*, *64*), providing a potential new source for lunar water. Beyond their roles in the exo-ionosphere, H^−^ ions may also influence the physical and chemical evolution of the surface itself. Considering the porous, castle-like structure of the lunar regolith (*65*), some H^−^ ions emitted from one grain can affect neighboring grains. Upon such impact, these ions, acting as strong electron donors, may inject electrons into mineral lattices. Their kinetic energy, together with near-surface electron capture into the lattice, could facilitate localized reduction distinct from solar wind protons (*66*, *67*) and may contribute to npFe^0^ formation.

Similar negative ions and the associated exo-ionospheres should also exist on other airless celestial bodies, including asteroids, icy moons, and Mercury (*17*). In regions farther from the Sun, such as the moons of Saturn and Jupiter, the weaker solar radiation would allow negative ions generated by surface interactions with surrounding plasma (including solar wind and magnetospheric particles) to persist for longer periods, which can lead to a higher density that can play more noticeable roles at those places.

## MATERIALS AND METHODS

### Instrumentation

The NILS instrument (*33*) onboard Chang’E-6 lander is a time-of-flight mass spectrometer, which is specifically designed to detect negative ions. An electromagnetic system is involved that can generate a variable magnetic field to suppress the entry of electrons. The instrument has an energy measurement range from 3 eV to 3 keV, divided into 48 channels, with a typical energy resolution of 14%. The instrument’s field of view is 120° wide vertically, with its boresight aligned horizontally with the ground. It has 16 angular pixels that are scanned sequentially. Particles of different masses can be distinguished by their different flight times within the time-of-flight system.

### Determination of the H^−^ energy spectrum

The raw NILS data consist of four dimensions: Time x Energy_bin x Viewing_direction x Mass_bin (*34*). We compiled all NILS monitoring intervals longer than 20 min, during which the internal high voltage remained at nominal levels. A total of six such intervals were identified. For each interval where the field of view was pointed below the horizon, the counts were accumulated, resulting in six integrated energy-mass matrices. To distinguish the different components within the energy-mass matrices, we adopted the code from Wieser *et al.* (*34*) and used a maximum likelihood fit method. The counts were assumed to be contributed by four components: electrons, negative hydrogen ions, negative oxygen ions, and accidental background counts. The time-of-flight response functions for electrons, hydrogen ions, and oxygen ions can be found in Canu-Blot *et al.* (*33*). Accidental background counts were assumed to be uniformly distributed across the mass bins. Note that in the fitting process, counts in the highest mass bin (number 63) were not considered. The confidence interval on the counts (subsequently converted to flux) is obtained via profile likelihood ratios (*68*). The conversion from counts to differential number flux was performed using equation 76 in Canu-Blot *et al.* (*33*), where the details of the derivation are provided. The resulting H^−^ energy spectrum data points with energies below the corresponding solar wind energy for each interval are shown in fig. S1, with examples for intervals 1 and 3 compared in Fig. 2 (A and B).

### Calculations of the integrated H^−^ flux and the average H^−^ energy

We calculate the integrated H^−^ flux by

$$F_{H^{-}}=\sum_{i}\frac{\left(E_{i+1}-E_{i}\right)}{2}\cdot \left(J_{i+1}+J_{i}\right)$$ (1)

and the average H^−^ energy by

$$E_{H^{-}}=\frac{\sum_{i}\frac{\left(E_{i+1}-E_{i}\right)}{2}\cdot \left(J_{i+1}+J_{i}\right)\cdot \frac{\left(E_{i+1}+E_{i}\right)}{2}}{\sum_{i}\frac{\left(E_{i+1}-E_{i}\right)}{2}\cdot \left(J_{i+1}+J_{i}\right)}$$ (2)

where $J_{i}$ is the differential flux at energy bin $E_{i}$.

The H^−^ signal only consistently exceeds the 90% significance limit curve at relatively higher energies and may be lower than the 90% significance limit curve at lower energies—particularly when the solar wind flux is low (Fig. 2A). Therefore, in our calculation for each interval, we selected the energy range from 200 eV up to the solar wind energy, including only those data points that exceeded the 90% significance limit curve.

### Calculations of the solar wind flux and the solar wind energy

With the solar wind velocity and the number density obtained from the ARTEMIS spacecraft, we calculated the solar wind normal flux by

$$F_{\text{sw},⊥}=n_{\text{SW}}\cdot \sqrt{V_{x,\text{sw}}^{2}+V_{y,\text{sw}}^{2}+V_{z,\text{sw}}^{2}}\cdot \text{cos}\left(\mathrm{SZA}\right)$$ (3)

and the solar wind energy by

$$E_{\text{SW}}=0.5\cdot m_{p}\cdot \left(V_{x,\text{sw}}^{2}+V_{y,\text{sw}}^{2}+V_{z,\text{sw}}^{2}\right)$$ (4)

where $n_{\text{SW}}$ is the solar wind density, SZA is the solar zenith angle, and $m_{p}$ is the proton mass. $V_{x,\text{sw}},V_{y,\text{sw}},V_{z,\text{sw}}$ are the solar wind velocity components along the *x*, *y*, and *z* directions, respectively. In this way, we calculated the average solar wind energy and the solar wind normal flux for each time interval.

### The fitting model for the H^−^ spectrum

Laboratory and theoretical studies have shown that the probability of particles being emitted from solid surfaces in a negatively charged state depends on the perpendicular component of the ejection velocity $V_{⊥}$ (*16*, *18*, *42*). During the short time that the emitted negative particle stays within a specific distance from the surface, the additional electron of the negative particle may return to the surface. The probability of a negative ion surviving from such a process is as follows

$$P^{-}∼\text{exp}\left(-\frac{\varphi_{w}-E_{A}}{{\mathrm{aV}}_{⊥}}\right)$$ (5)

where $\varphi_{w}$ represents the surface work function, $E_{A}$ is the electron affinities, and *a* is a constant dependent on the specific particle-surface combination. It is sometimes more convenient to write the exponential dependence in the following form (*69*, *70*)

$$P^{-}∼\text{exp}\left(-\frac{C_{V}}{V_{⊥}}\right)$$ (6)

where $C_{V}$ is the velocity-dependence parameter.

Futaana *et al.* (*39*) analyzed the ENA data from Chandrayaan-1 spacecraft and found that the ENA energy approximately followed a Maxwell-Boltzmann distribution, which can be expressed by the following empirical formula

$$f\left(E_{e}\right)∼F_{\text{sw},⊥}\frac{E_{e}}{{\left(\mathrm{kT}\right)}^{2}}\text{exp}\left(-\frac{E_{e}}{\mathrm{kT}}\right)$$ (7)

$$T\;\left[\text{in}\;K\right]=V_{\text{SW}}\left[\text{in}\;m/s\right]\times 3.17-2.31\times 10^{4}$$ (8)

where *k* is the Boltzmann constant, $V_{\text{SW}}$ is the solar wind velocity, *T* is the ENA characteristic temperature in the unit of kelvin, and $E_{e}$ is the ejection energy. We incorporate the ejection velocity dependency into the empirical formula for ENA scattering on the lunar surface, to fit the energy spectrum of the scattered H^−^ ions. Then, we obtain an empirical model of the H^−^ spectrum as follows

$$f\left(E_{e}\right)=AF_{\text{sw},⊥}\frac{E_{e}}{{\left(\mathrm{kT}\right)}^{2}}\text{exp}\left(-\frac{E_{e}}{\mathrm{kT}}\right)\text{exp}\left(-\frac{C_{V}}{V}\right)$$ (9)

where $C_{V}$ is the velocity dependence fitting parameter, and *A* is the fitting parameter related to the scattering yield. Note that the surface in the laboratory is flat, in contrast to the lunar soil, which is rough and castle-like with porous structures. The actual emission direction of negative ions from the lunar surface is widely distributed, and hence we use the H^−^ velocity measured by the NILS instrument to represent the ejection velocity, which is known as *V* in Eq. 9. Consequently, the observed energy spectrum points serve as the input data for the least squares method, which we applied to obtain the fitting lines of the H^−^ spectrum for each interval. This empirical formula was also used by Lue *et al.* (*48*) to achieve a good fit for the H^+^ energy spectrum of lunar surface scattering.

### Distribution of H^−^ ions around the Moon

The location, angle, and velocity of each particle ejected from the surface, as well as its lifetime with the effect of photodetachment, are obtained through Monte-Carlo random sampling. For example, we generate a random number *R* between 0 and 1 and then invert the distribution function to determine the ejection velocity

$$R\left(0\to 1\right)=\frac{\int_{0}^{V}\frac{E_{e}}{{\left(\mathrm{kT}\right)}^{2}}\text{exp}\left(-\frac{E_{e}}{\mathrm{kT}}\right)\text{exp}\left(-\frac{C_{V}}{V}\right)\mathrm{dV}}{\int_{0}^{V_{\text{SW}}}\;\frac{E_{e}}{{\left(\mathrm{kT}\right)}^{2}}\text{exp}\left(-\frac{E_{e}}{\mathrm{kT}}\right)\text{exp}\left(-\frac{C_{V}}{V}\right)\mathrm{dV}}$$ (10)

On the dayside, we adopt the angular distribution function of scattered H ENAs from Chandrayaan-1 (*50*) to describe the emission angles of H^−^ ions, due to the limited angular coverage of NILS. On the nightside, where the solar wind incidence angle cannot be determined (*55*), the emission directions of H^−^ ions are modeled with a uniform angular distribution. For each ejected particle, it has a unique weight determined by its yield, which can be calculated by multiplying the solar wind normal flux at each location by the scattering yield. In the test particle simulation, we consider the electromagnetic forces derived from the hybrid model, as well as the gravitational force of the Moon, in the particle dynamics. We use the fourth-order Runge-Kutta method to calculate the time advance of the position (*X*, *Y*, and *Z*) and velocity (*Vx*, *Vy*, and *Vz*) of the particles. The dayside density can reach an equilibrium in 0.7 s, while the nightside density continues to extend as a long tail. We chose a terminal time of 50 s to obtain a limited-length tail on the nightside, as shown in Fig. 3.

### Hybrid model

The hybrid model uses the FLASH software (*71*). See Holmstrom (*51*) and references therein for full details of the model. Solar wind protons are injected into the simulation domain at the upstream inflow boundary and are removed if they reach the inner boundary, a sphere of radius 1730 km. The electric and magnetic fields are solved for in all of the simulation domain. The resistivity is 10^7^ Ω·m inside the inner boundary, zero in the solar wind plasma, and 10^7^ Ω·m in vacuum regions. The cell size is 80 km and the time step is 0.001 s. The hybrid simulations are run until a steady state at 28 s. We can note that the hybrid model assumes that all negative charged particles are the mass-less electrons. Thus, the hybrid model is not suitable if negative ions are a significant fraction of the plasma density. In such cases, a full PIC model, with electrons and negative ions represented as particles, would be more suitable. Full global modeling of the interaction between the Moon and the solar wind would however be computationally infeasible for such a model. Consequently, here, we still choose a hybrid model to provide the background fields for the test-particle simulation.
