# Supplementary material for: Chang’E-6 reveals solar wind–dependent H− ions on the Moon
Source: Sci Adv. 2026 Mar 13;12(11):eadw1162. doi: 10.1126/sciadv.adw1162 (PMC12985742; doi:10.1126/sciadv.adw1162)
Supplement: Supplementary file 1 — Figs. S1 to S5 Table S1 [file sciadv.adw1162_sm.pdf]

Science Advances
AAAS

Supplementary Materials for

Chang'E-6 reveals solar wind–dependent H^- ions on the Moon

Tianhua Zhong *et al.*

Corresponding author: Lianghai Xie, xielianghai@nssc.ac.cn; Aibing Zhang, zhab@nssc.ac.cn; Martin Wieser, martin.wieser@irf.se

Sci. Adv. **12**, eadw1162 (2026)
DOI: 10.1126/sciadv.adw1162

This PDF file includes:

Figs. S1 to S5
Table S1

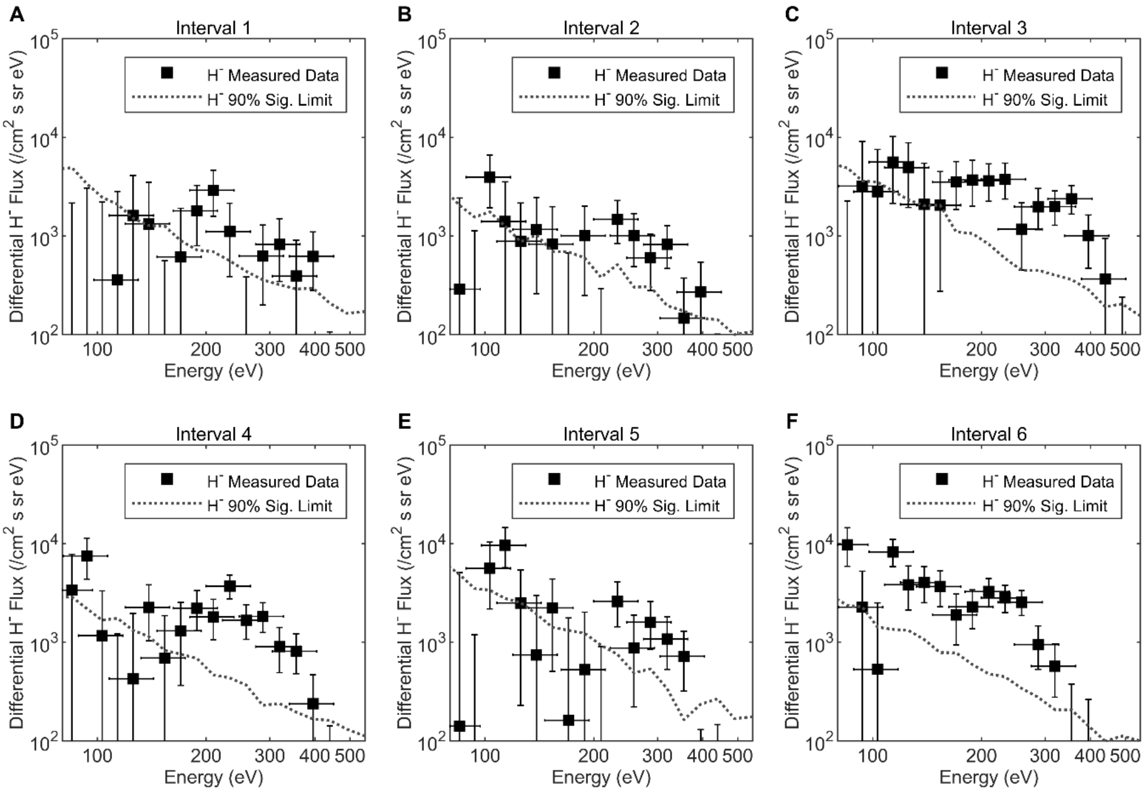

Fig. S1. H⁻ spectra obtained during six NILS observation intervals.

(**A** to **F**) Differential energy spectra of H⁻ ions measured by the NILS instrument during six effective observation intervals. The black squares represent the measured data points, and the vertical error bars represent 68% confidence intervals. The horizontal error bars represent the energy resolution of the instrument. Confidence intervals without a data point indicate that the data point is below the differential flux range shown. The dotted lines correspond to a 90% significance limit, below which the differential flux cannot be significantly distinguished from zero.

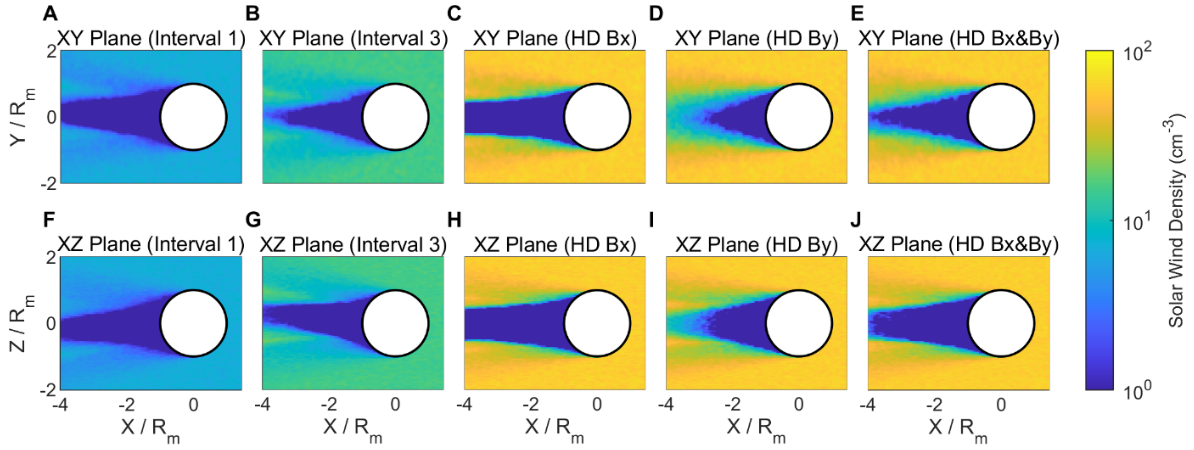

Fig. S2. Hybrid simulation results of the solar wind proton number density in different planes.

(**A** to **E**) Density distributions of solar-wind protons in the XY planes for interval 1, interval 3, and the three extremely high solar wind density cases (HD Bx, HD By, and HD Bx&By), respectively. (**F** to **J**) Density distributions of solar-wind protons in the XZ planes for interval 1, interval 3, and the three extremely high solar wind density cases (HD Bx, HD By, and HD Bx&By), respectively.

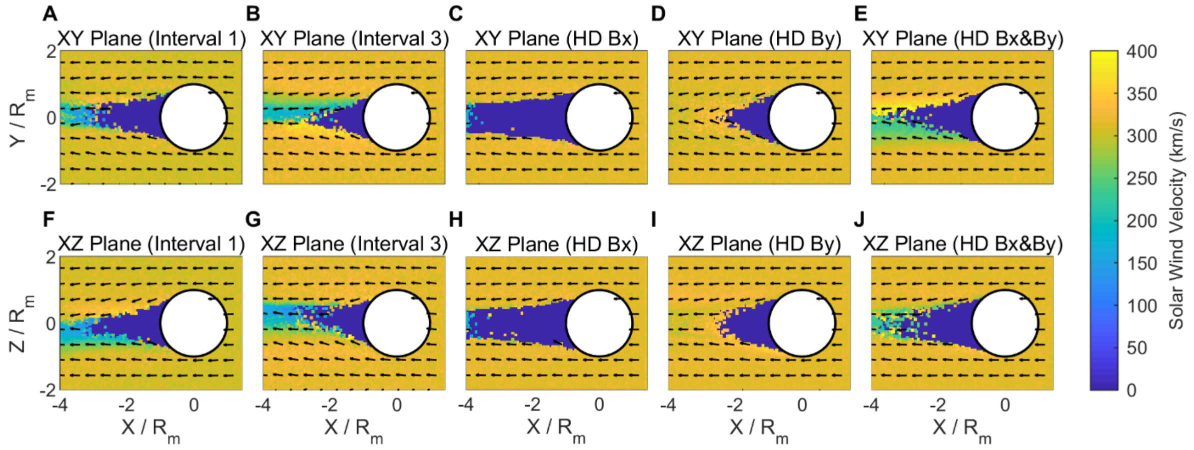

Fig. S3. Hybrid simulation results of the solar wind velocity in different planes.

(**A** to **E**) Velocity distributions of solar wind protons in the XY planes for interval 1, interval 3, and the three extremely high solar wind density cases (HD Bx, HD By, and HD Bx&By), respectively. (**F** to **J**) Velocity distributions of solar wind protons in the XZ planes for interval 1, interval 3, and the three extremely high solar wind density cases (HD Bx, HD By, and HD Bx&By), respectively. The black dashed arrows indicate the velocity directions.

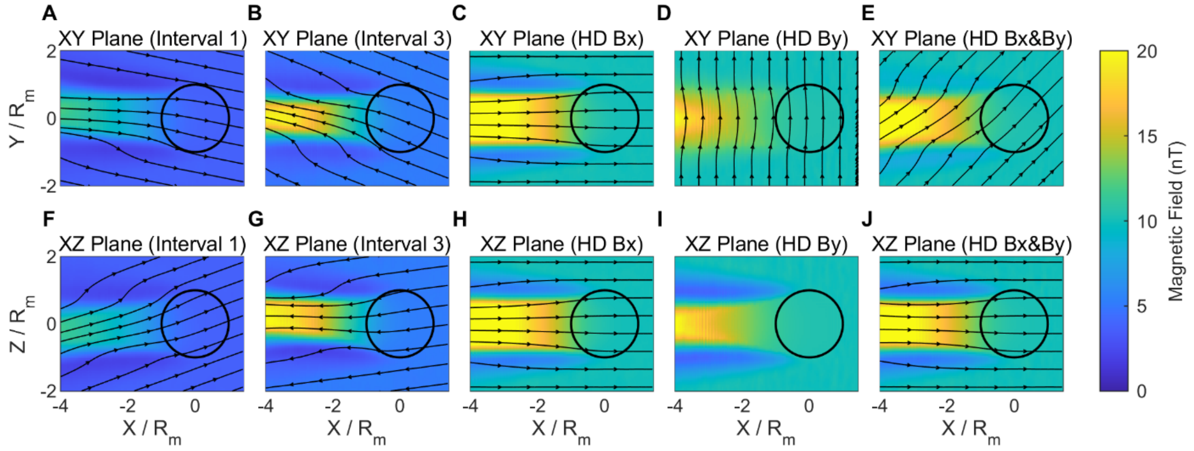

Fig. S4. Hybrid simulation results of the magnetic field strength in different planes.

(**A** to **E**) Magnetic field distributions in the XY planes for interval 1, interval 3, and the three extremely high solar wind density cases (HD Bx, HD By, and HD Bx&By), respectively. (**F** to **J**) Magnetic field distributions in the XZ planes for interval 1, interval 3, and the three extremely high solar wind density cases (HD Bx, HD By, and HD Bx&By), respectively. The black lines with arrows indicate the magnetic field lines. For the panels without arrows, the magnetic field is essentially perpendicular to the plane.

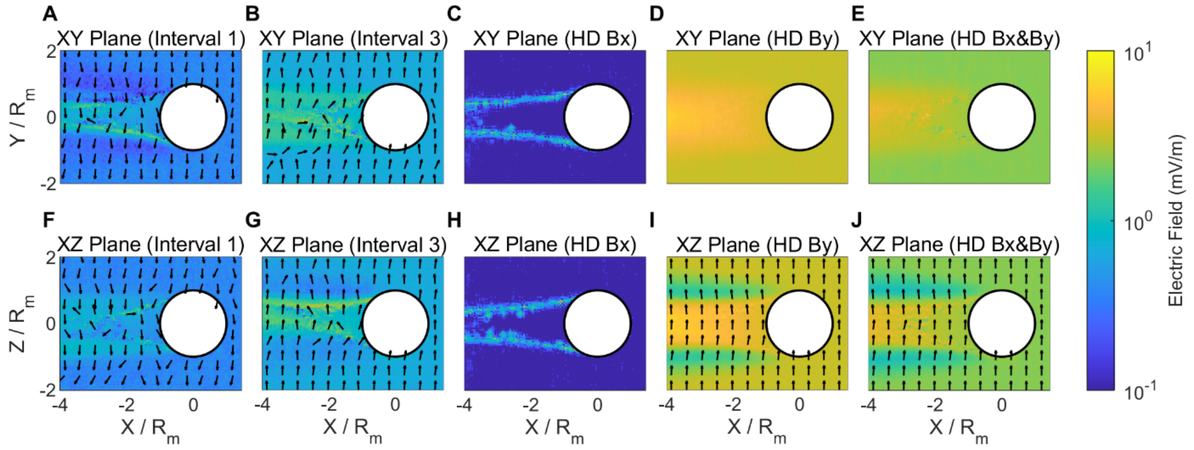

Fig. S5. Hybrid simulation results of the electric field strength in different planes.

(**A** to **E**) Electric field distributions in the XY planes for interval 1, interval 3, and the three extremely high solar wind density cases (HD Bx, HD By, and HD Bx&By), respectively. (**F** to **J**) Electric field distributions in the XZ planes for interval 1, interval 3, and the three extremely high solar wind density cases (HD Bx, HD By, and HD Bx&By), respectively.The black dashed arrows indicate the electric field directions. For the panels without arrows, the electric field is essentially oriented perpendicular to the plane.

Interval	Date	Start Time (UTC)	End Time (UTC)	Average solar wind density (cm⁻³)	Average solar wind energy (eV)
1	June 2	03:01:58	03:24:47	6.91	485.04
2	June 2	06:20:13	07:05:20	8.1	469.75
3	June 2	17:14:06	17:38:24	15.38	518.97
4	June 2	19:07:00	20:00:13	14.96	474.94
5	June 2	00:44:07	01:07:35	10.48	462.11
6	June 3	02:41:44	03:37:35	10.87	449.38

Table S1. NILS observation intervals and corresponding solar wind parameters.
Six effective observation intervals, during which reliable H^- spectra were obtained, are listed with their start and end times (UTC), and the corresponding average solar wind proton density and energy measured by the ARTEMIS spacecraft.
